# Innovative Approaches to Address the Technical Challenge of Hernial Sac Distension Due to Pneumoperitoneum in the Synchronous Management of Complex Hernias in Individuals With Morbid Obesity

**DOI:** 10.7759/cureus.59897

**Published:** 2024-05-08

**Authors:** Ankita Bajpai, Akshay Anand, Awanish Kumar, Tirushi Jain, Ajay K Pal, Amit Karnik, Harvinder S Pahwa, Abhinav A Sonkar

**Affiliations:** 1 Surgery, All India Institute of Medical Sciences, New Delhi, New Delhi, IND; 2 Surgery, King George’s Medical University, Lucknow, IND; 3 General Surgery, King George’s Medical University, Lucknow, IND

**Keywords:** complex hernia, hernia in obese, technical challenge, morbid obesity, ventral hernia

## Abstract

Although repairing ventral hernias in individuals who have undergone bariatric surgery is a common practice, persistent technical intricacies and controversies surround their management. Concurrently, addressing ventral hernias in morbidly obese patients undergoing bariatric surgery presents a significant surgical challenge, amplified by the larger intraperitoneal cavities and the presence of large hernial sacs. This technical report introduces two innovative techniques to alleviate the challenge of hernia sac distension due to pneumoperitoneum associated with simultaneous bariatric surgery and ventral hernia repair using laparoscopic technique. The methods are designed to address the complexities of the procedures, making their simultaneous execution feasible and safe. The goal is to eliminate the need for two separate interventions while ensuring the outcomes of each procedure remain uncompromised. The larger intraperitoneal cavities and the presence of large hernial sacs are managed successfully, demonstrating the feasibility and safety of the introduced methods. Importantly, the simultaneous execution of both procedures does not compromise the outcomes of either intervention.

Concurrently managing ventral hernias in morbidly obese patients undergoing bariatric surgery requires innovative solutions to overcome technical challenges. The introduction of these two novel techniques proves to be a valuable approach, making simultaneous execution feasible and safe. Eliminating the need for two separate interventions streamlines the surgical process without compromising the outcomes of either bariatric surgery or ventral hernia repair.

## Introduction

The prevalence of obesity continues to rise worldwide and has already reached pandemic proportions [1-3]. Bariatric surgery (BS) has proven to be highly efficacious in treating obesity and its comorbidities and has rapidly become a treatment option for severe obesity [2-5]. In terms of surgical intervention, three procedures mainly comprise the major bariatric volume across the globe. The common procedures include sleeve gastrectomy (SG), Roux-en-Y gastric bypass, and adjustable gastric banding, with SG being the most popular bariatric operation [3-6]. Ventral hernia (VeH) is a common comorbidity seen in patients with obesity compared to the non-obese population. Obesity can itself be a causative factor toward primary or recurrent VeH [7-10]. VeH with huge hernia sacs can be challenging to treat due to ergonomics. Simultaneously managing VeHs in morbidly obese patients undergoing bariatric surgery presents a notable surgical hurdle. The expanded intraperitoneal cavities in obese individuals, combined with the presence of sizable hernial sacs, compound these ergonomic difficulties. We report two novel methods to ease the surgical procedure of concomitant laparoscopic SG with intraperitoneal onlay mesh (IPOM) repair in morbidly obese patients. These innovative methods not only enable the safe and feasible simultaneous execution of both procedures, especially addressing the intraoperative surgical challenge posed by hernia sac distension, but also eliminate the necessity for two separate interventions, all the while ensuring that the outcomes of either procedure remain uncompromised.

## Technical report

Case 1

Description

**Figure 1:**
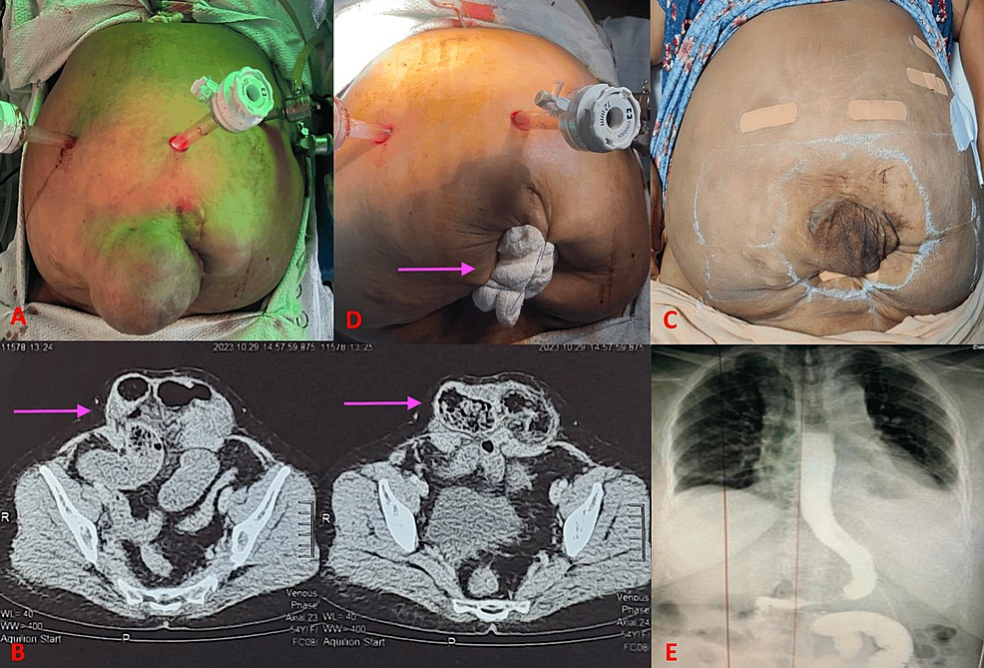
(A) The patient with an infraumbilical incisional hernia with a large sac. (B) CT scan showing the abdominal defect in the lower midline (pink arrows). (C) Port-site positions (taped). (D) Surgical mop plugged and tied with temporary sutures (pink arrow). (E) Gastrograffin X-ray imaging to delineate the post-procedure gastrointestinal anatomy.

A 51-year-old woman presented to King George’s Medical University, Department of General Surgery, Bariatric Unit Outpatient Department (OPD) with an infraumbilical hernia for four months. The patient had undergone lower segment cesarean section four times with the last one 12 years ago. On presentation to us, she had an infraumbilical incisional hernia with a 4 cm midline facial defect below the umbilicus. The hernial sac was quite large with redundant skin (Figure 1, Panel A). At the time of presentation, her weight was 84 kg and height 1.42 m, corresponding to severe (morbid) class III obesity [11], with a calculated body mass index (BMI) of 41.5 kg/m². Medical comorbidities included severe obstructive sleep apnea (OSA) and osteoarthritis (OA). As the patient had VeH and was morbidly obese with OSA and OA, she was offered combined BS with hernia repair. Abdominal CT was performed and the size of the defect was approximated to be 44 mm in the infraumbilical region (Figure 1, Panel B). After completing preoperative evaluation and education, a laparoscopic SG combined with IPOM+ hernia repair was done.

Surgical Technique for Bariatric Procedure

Before the operation, the patient received a single-dose antibiotic during induction, and a 32 Fr orogastric bougie was orally introduced and positioned a few centimeters below the gastroesophageal transition. Anti-embolic precautions were implemented through the attachment of sequential compression devices. The procedure was conducted with the patient in a supine position, legs split, and in a reverse Trendelenburg position. The primary surgeon stood between the lower limbs, while the assistant surgeon was positioned on the right side. Pneumoperitoneum was established using a Veress needle at Palmer’s point, with an inflation pressure of 14 mmHg and a flow of up to 50 L/minute of carbon dioxide.

The initial port (10 mm) was placed at the horizontal level of the left midclavicular line, near the costal margin (Palmer’s point) for the initial entry. Subsequent ports were positioned under optical visualization. The second 5 mm port was placed in the left anterior axillary line along the costal margin. The third 12 mm port was inserted, using the xiphoid-umbilical line as a reference, at the junction of two-thirds with a lower third. The fourth trocar (12 mm) was positioned on the right side of the patient at the right midclavicular line (Figure 1, Panel C). Additionally, a Nathanson retractor was deployed in the epigastric region for liver retraction. SG was performed using the conventional technique.

Difficulty During SG and Novel Intervention

The large hernial sac below the umbilicus expanded upon pneumoperitoneum creation, causing frequent loss of pneumoperitoneum and complicating the SG procedure. To address this issue, the surgical team deflated the abdomen and placed a surgical abdominal mop externally onto the defect area, securing it with sutures. This mop plug effectively prevented sac distension during the pneumoperitoneum creation during the SG (Figure 1, Panel D). After SG, fat dissection was performed to create space for mesh placement. A 15 cm × 20 cm mesh was inserted and secured with transfascial sutures and a mesh fixation device, closing the umbilical defect (IPOM+ procedure).

After ensuring hemostasis and completing the World Health Organization (WHO) surgical safety checklist, the remaining trocars were removed under visualization to prevent port-site bleeding. Subcutaneous sutures with Monocryl 3-0 (polydioxanone) were used to close the skin. Following institutional protocol, the patient began oral clear liquids eight hours after surgery (Figure 1, Panel E) and was discharged on the seventh postoperative day. Notably, there were no postoperative complications.

Case 2

Description

**Figure 2:**
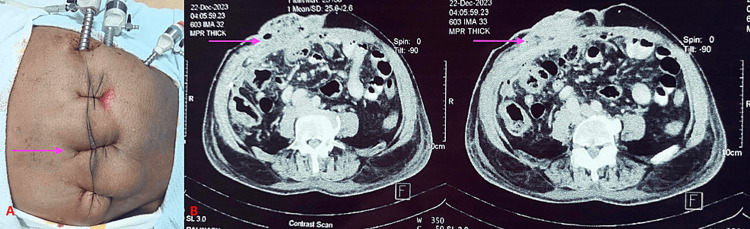
(A) The patient with a right subcostal incisional hernia defect closed with a transabdominal suture (Nylon no. 1 suture) (pink arrow), (B) CT scan showing the abdominal defect in the right subcostal region post-open cholecystectomy (pink arrows).

A 48-year-old morbidly obese female visited the Bariatric Unit OPD with a post-cholecystectomy incision hernia persisting for a year. Upon examination, a fascial defect of approximately 43 mm in the right subcostal region was noted. She had a BMI of 39.7 kg/m², and her medical history included diabetes mellitus. The option of combined bariatric surgery along with hernia repair was presented to her. An abdominal CT scan revealed a 48 mm defect in the right subcostal region (Figure 2, Panel A). Following thorough preoperative assessment and education, a laparoscopic SG was performed in conjunction with IPOM+ repair.

Difficulty During the Procedure and Novel Intervention

The surgical team encountered a similar difficulty during the bariatric procedure when dealing with the right subcostal hernial sac, resulting in inadequate pneumoperitoneum. To address this challenge, after reducing the contents and adhesiolysis, the operating team placed three to four transabdominal sutures across the abdominal defect (Figure 2, Panel B). The defect was then primarily closed to overcome the surgical challenge of intraoperative working space. Following the completion of SG, the sutures were released and the IPOM procedure was performed. A 15 cm × 15 cm mesh was placed and secured using intracorporeal sutures and a mesh fixation device. Notably, no complications were noted in the postoperative period.

## Discussion

VeH is relatively prevalent in the morbidly obese population, primarily due to elevated intra-abdominal pressures compared to individuals with a normal weight. This is compounded by the weakening of the abdominal wall musculature [11]. Identifying and addressing these occurrences are essential for improving patient outcomes, particularly given the elevated incidence of VeH, reaching up to 20%, commonly observed in morbidly obese individuals. Contributing factors encompass a greater volume of visceral fat, increased intra-abdominal pressure, a larger abdominal wall perimeter, an amplified risk of surgical site infection, and related comorbidities such as diabetes. The simultaneous undertaking of BS and VeH repair introduces technological challenges, especially when confronted with incarcerated VeHs that restrict the operative field for bariatric procedures and large hernial sacs which are often a reason for the loss of adequate intraperitoneal working field.

Obesity is acknowledged as a risk factor for the emergence of accompanying conditions, including cardiovascular disease, type 2 diabetes mellitus, malignancies, asthma, OA, chronic back pain, OSA, non-alcoholic fatty liver disease, and gallbladder ailments [12]. Patients with obesity and VeH who display specific characteristics, including a BMI below 50 kg/m², a gynecoid body physique, midline reducible hernias, abdominal wall thickness less than 4 cm, and a maximum defect diameter not exceeding 8 cm, are classified as having a favorable profile. Choosing a bariatric approach presents a challenging subgroup among these individuals, introducing heightened intra and postoperative complexities and, consequently, elevated mortality and morbidity rates [6]. There is a debate regarding whether concomitant BS and VeH repair might entail more complications compared to BS alone [13]. Recognizing the significance of performing both procedures concurrently is apparent, as the alternative, i.e., postponing VeH repair after BS, may decrease the likelihood of recurrence and simplify hernia repair but is linked to a higher incidence of unresolved hernia complications [14]. Comprehensive studies have scrutinized complications associated with simultaneous BS and VeH repair [15]. In one of the largest series (n = 503), Spaniolas et al. [8] suggested that synchronous VeH repair is only linked to an elevated surgical-site infection rate, not overall morbidity. Sharma et al., in a series (n = 159) comparing techniques (open vs. laparoscopic hernia repair, mesh vs. primary suture), disclosed a minimal early complication rate, indicating the feasibility of synchronous repair. However, a 12-month follow-up revealed a moderately high hernia recurrence rate (25%), necessitating reoperation [16]. Various studies have reported diverse recurrence rates after simultaneous VeH repair and BS in the overall obese population. Ching et al. [17] reported a 12% recurrence rate (n = 168), Krecioch et al. [18] reported 7.8% (n = 144), and Raziel et al. [19] reported 1.8% (n = 54). Smaller cohort studies [9,19,20] concurred that VeH repair during BS is feasible and concluded that concurrent surgery is safe, without impeding the outcomes of the bariatric procedure. We are unable to provide information on the actual recurrence rate, as neither patient has reported any recurrence within the last 11 months and 15 months for case 1 and case 2, respectively. A shorter follow-up period represents a constraint in this report. However, the primary focus of this study lies in delineating techniques aimed at surmounting the intraoperative surgical challenge posed by the loss of pneumoperitoneum in the distended hernia sac and the constrained working space.

The primary limitation of a staged repair lies in the risk of hernia-related complications, such as incarceration and strangulation, with reported incidence rates ranging from 3.7% to 35% [13,21]. This variability appears to be associated with the size of the hernia defect, with higher rates observed for smaller defects. Consequently, some studies recommend addressing small defects simultaneously during the bariatric procedure while adopting a staged approach for larger ones [13]. In comparison to open techniques, laparoscopic VeH repair consistently exhibits lower overall complication rates, shorter hospital stays, and faster return to work. Existing literature predominantly indicates slightly lower recurrence rates in laparoscopic repair, although this association has not always reached statistical significance. Despite its advantages, laparoscopy is not without drawbacks, including a heightened risk of visceral injury and increased technical difficulty due to the compromised intraperitoneal space [22].

From a technological perspective, the authors have demonstrated that closing the fascial defect is far simpler with these novel methods. After the BS is completed, the laparoscopic VeH repair can be performed. Due to the possibility of maintaining smaller incisions, the advantages of laparoscopy are maintained.

## Conclusions

VeHs are a common occurrence in individuals undergoing bariatric procedures. The heightened risk of severe complications linked to leaving a VeH untreated after BS has spurred a growing consensus in favor of addressing both issues simultaneously. The comparatively low occurrence of complications associated with VeH repair, encompassing issues such as recurrence, hematoma, infection, and seroma, coupled with the benefits of avoiding an additional surgical procedure, underscores the viability of concurrent repair. As a result, the approach of performing both BS and VeH repair within a dedicated bariatric unit emerges as a practical and feasible strategy, featuring unique methods to alleviate intraoperative challenges.
